# Enhanced tumor suppression in colorectal cancer via berberine-loaded PEG-PLGA nanoparticles

**DOI:** 10.3389/fphar.2024.1500731

**Published:** 2024-11-01

**Authors:** Fei Shen, Yun-Sheng Zheng, Lan Dong, Ziyang Cao, Jie Cao

**Affiliations:** ^1^ Department of General Surgery, The First Affiliated Hospital, Jinan University, Guangzhou, China; ^2^ Department of General Surgery, Guangzhou Digestive Disease Center, The Second Affiliated Hospital, School of Medicine, South China University of Technology, Guangzhou, China

**Keywords:** colorectal cancer, berberine, RNA-seq, nanocarrier, signaling pathways

## Abstract

Colorectal cancer (CRC) stands as the third most widespread cancer globally with poor prognosis. Berberine (Ber), as one herbal phytochemical, showed promise in CRC therapy, but its exact mechanism is unclear. Small molecule traditional drugs face challenges in quick metabolism and low bio-availability after systemic administration. Nanodrug deliver system, with their unique properties, has the advantages of protecting drugs, improving drug bio-availability, and reducing toxic and side effects, which exhibited huge drug delivery potential. Herein, the PEG-PLGA nanocarrier was used for encapsulated Ber according to nanoprecipitation and obtained nanomedicine, denoted as NPBer. *In vitro*, the flow cytometry test and CCK8 assays indicated that NPBer was more easily taken up by HCT116 CRC cells, and had stronger inhibition on cell proliferation with the increase of drug concentration. In addition, RNA-Seq was employed to explore the alterations in the transcriptomes of cancer cells subsequent to treatment with Free Ber or NPBer.The sequencing results indicate that Free Ber could activate cellular aging mechanisms, intensified the iron death pathway, optimized oxidative phosphorylation efficiency, exacerbated apoptosis, accelerated programmed cell death, and negatively modulated key signaling pathways in CRC cells including Wnt, TGF-beta, Hippo, and mTOR signaling pathways. Based on PEG-PLGA nanocarriers, NPBer can improve the *in vivo* delivery efficiency of Ber, thereby enhancing its antitumor efficacy *in vivo*, enhancing apoptosis by enhancing the mitochondrial autophagy and autophagy activities of CRC cells, negatively regulating the inflammatory mediator to regulate TRP channels, and inhibiting the activation of Notch signaling pathway. *In vivo*, NPBer can significantly improve its accumulation and durable drug targeting in tumor site, resulting in induce maximum cell apoptosis and effectively inhibit the proliferation of HCT116 tumor. This strategy provided a promising antitumor therapeutic strategy using Ber-based drugs.

## 1 Introduction

Colorectal cancer (CRC) is classified as a malignant tumor of the digestive system and ranks third in the estimated number of new cases among both men and women in the United States (Siegel et al., 2024), also seriously endangering the health of the Chinese people (Ju et al., 2023). Surgical treatment, chemotherapy, radiotherapy, and immunotherapy are currently the main treatments for CRC (Shinji et al., 2022; Riesco-Martinez et al., 2022; Modest et al., 2019; Sanuki et al., 2022; Zhao et al., 2022). Among these treatments, chemotherapy plays a crucial role as both a primary and adjuvant therapy for CRC. Current research focuses on optimizing the efficacy of chemotherapeutic agents, while addressing challenges related to drug toxicity, side effects, and the development of resistance (Gottesman et al., 2023; Yang et al., 2024). Most diseases are caused by abnormal gene expression, and identifying drugs that can specifically target these genes is crucial for disease prevention and treatment (Singh et al., 2018; Tao et al., 2024). It is worth noting that traditional Chinese medicine (TCM) has seen increasingly widespread application in tumor treatment (Xiang et al., 2019; Liu Y. et al., 2024). Numerous studies have consistently demonstrated that TCM not only has the potential to alleviate cancer-related symptoms, such as fatigue, loss of appetite, cachexia, and persistent pain, thereby improving patients’ quality of life, but it also effectively reduces the adverse reactions and complications commonly associated with chemotherapy, radiotherapy, and targeted therapies (Zhou and Jiang, 2019; Zhang et al., 2021; Huang et al., 2019; Liu K. et al., 2024; Wang et al., 2022). However, the poor water solubility and low bio-availability of many TCM compounds, along with their short circulation times in the body, result in reduced drug efficacy and limited accumulation in tumor tissues. Thus, enhancing the therapeutic potency of chemotherapeutic agents and improving their tumor accumulation and retention is a key. Recently, nanocarriers have emerged as important tools in drug delivery, regarded as ideal vehicles for transporting a wide range of drugs due to their excellent storage stability, inherent biocompatibility, biodegradability, and ease of surface modification (Sun et al., 2022; Wei et al., 2022; Kang et al., 2023). Furthermore, novel strategies employing nanocarriers have shown promise in overcoming drug resistance in cancer (Hu et al., 2022; Benko et al., 2021).

In this study, we utilized a FDA-proved nanomaterial, PEG-PLGA (Zhang et al., 2014), consist of covalent connected polyethylene glycol (PEG) and PLGA, to encapsulate Berberine (Ber) according to nanoprecipitation and obtained a berberine-loaded nanomedicine (denoted as NPBer). Flow cytometry and confocal microscopy revealed that NPBer is effectively internalized by HCT116 tumor cells, leading to significant inhibition of HCT116 CRC cell proliferation. Furthermore, to assess its *in vivo* antitumor efficacy, the HCT116 nude mouse xenograft model was used to study the systemic administration of NPBer. The results demonstrated that NPBer could effectively accumulate and remains in the tumor site, ultimately achieving notable anti-tumor effects. These findings provided a novel nanocarrier-based delivery strategy for Ber drug therapy in CRC.

## 2 Materials and methods

### 2.1 Cell lines and animals

The HCT116 CRC cancer cell lines were sourced from the American Type Culture Collection and then propagated in Dulbecco’s Modified Eagle Medium (DMEM) that was fortified with 10% fetal bovine serum (FBS) and 1% penicillin-streptomycin, maintained at 37°C in an incubator, which was adjusted to a 5% CO_2_ environment.

The male BALB/c nude mice (aged 6 weeks) were procured from Charles River, Beijing, China. The experimental procedures received approval from the Animal Care and Use Committee of the South China University of Technology.

### 2.2 Preparation of NPBer

PEG-PLGA nanoparticles and Berberine (Ber, Sigma-Aldrich, Germany) were fabricated according to the nanoprecipitation method (Almoustafa et al., 2017). After uniformly mixing 10 mg of polyethylene glycol-poly lactic acid-co-glycolic acid (PEG-PLGA) (1 mL DMSO) with 1 mg of Ber (200 µL DMSO), the mixture was added dropwise to 1 mL of deionized water and stirred for 2 h. Free drugs and DMSO were removed by dialysis. The particles were concentrated using a rotary evaporator, and the drug concentration was quantified using a microplate reader (UV absorption peak at 340 nm) to prepare the nanoparticle Ber (NPBer). Therefore, the drug loading capacity (DLC) of Ber within NPBer was quantified by applying the subsequent equation: DLC% = (M_Ber_/M_nanocarrier_) × 100%.

### 2.3 *In Vitro* cytotoxicity evaluation using CCK-8 assay

To examine the effect of PEG-PLGA nanomaterials on cell proliferation *in vitro*, the CCK-8 reagent (supplied by Biosharp, Hefei, China) was employed. The experimental protocol commenced by seeding HCT116 CRC cells onto 96-well plates at a density of 6 × 10³ cells per well. These plates were then incubated at 37°C to allow for cellular attachment and growth.The culture medium was exchanged with complete medium, which contained PEG-PLGA at varying concentrations. The CCK-8 reagent was administered to each group according to the manufacturer’s instructions. The absorbance was then measured using an appropriate spectrophotometer to quantify the viability of HCT116 CRC cells.

Further, in order to verify whether Ber can be rapidly absorbed by cells and continue to act after being coated with nanomaterials. Subsequently, after cell adhesion, the culture medium was exchanged with complete medium, which contained different concentrations of Free Ber, or NPBer for 4 h, replaced the complete medium, and continued to incubate for 20 h, and then carried out CCK-8 detection.

To assess the ongoing cytotoxic effects of Free Ber or NPBer on HCT116 CRC cells, after cell adhesion, the culture medium was exchanged with complete medium, which contained either Free Ber, or NPBer, each at varying concentrations, and carried out CCK-8 detection after a 36-h incubation period.

### 2.4 *In Vitro* cellular uptake

To evaluate the cellular internalization of the nanoparticles, both confocal laser scanning microscopy (CLSM) and flow cytometry techniques were utilized.

In the flow cytometry experiment, the initiation of the experimental protocol involved seeding HCT116 CRC cells onto 6-well plates, ensuring a density of 1 × 10^5^ cells per well, and then subsequent to the incubation with PBS, Free Ber or NPBer for 2 h or 4 h. The resulting cell suspension was then analyzed using a flow cytometer to quantify the uptake of the nanoparticles.

In the CLSM protocol, HCT116 CRC cells were plated onto 12-well plates equipped with cell-adherent slides. Once the cells adhered, the culture medium was exchanged with a serum-free medium containing Free Ber or NPBer. Following a 4-h incubation period, the cells underwent a rinsing process with phosphate-buffered saline (PBS), followed by fixation with 4% paraformaldehyde (PFA). Subsequently, the cells were counterstained with Hoechst 33,342 living cell staining solution (Beyotime, China) to visualize their nuclei, and subsequently observed under a confocal laser scanning microscope.

### 2.5 Transcriptomic analysis for uncovering anticancer mechanisms of NPBer

To elucidate the underlying anticancer biological mechanisms of NPBer, we conducted a comprehensive transcriptomic analysis on cancer cells utilizing RNA sequencing technology.

### 2.6 Biodistribution and targeting capacity of NPBer *In Vivo*

HCT116 CRC tumor-bearing mice were intravenously injected with Free Ber or NPBer (Ber = 1.0 mg/kg, n = 3). 24 h after drug injection, the mice were humanely euthanized, and subsequently, their tumor tissues and primary organs were harvested, and the Xenogen IVIS Lumina system (Caliper Life Sciences) was utilized to capture images of the Mean Fluorescence Intensity (MFI) for further imaging analysis.

### 2.7 *In Vivo* antitumor activity of NPBer

A total of 15 Tumor-bearing Balb/c mice were randomly allocated into groups of five mice each, and underwent treatment via tail vein injection. The treatments included PBS, Free Ber and NPBer (at a concentration of 1.0 mg/kg). Throughout the experiment, the tumor volume and body weight of the mice were carefully monitored and recorded.

Following a period of 15 days, the mice underwent euthanasia procedures. Subsequently, their tumors were carefully excised and then precisely weighed. Additionally, the vital organs including the heart, liver, spleen, lungs, and kidneys were harvested and subjected to H&E staining for histological examination. Blood samples were also collected for serum biochemical analysis. Alanine aminotransferase (ALT/GPT) test kit, creatinine (Cr) test kit, urea nitrogen (BUN) test kit, uric acid (UA) test kit, albumin test kit, aspartate aminotransferase (AST/GOT) test kit were purchased from Nanjing Jianguo Bioengineering Research Institute Co., LTD., and detected by ELISA method according to instructions in the manual.

For the tumor tissues collected from the euthanized mice, formalin fixation was carried out to prepare paraffin-embedded slides, which were subsequently subjected to TUNEL assay (#G1504, Servicebio, China) for detecting cell apoptosis and Ki67 (#GB111141, Servicebio, China) immunohistochemistry staining for the assessment of proliferation.

### 2.8 Statistical analysis

All data are expressed as mean ± standard deviation. SPSS 18.0 software was used for statistical analysis. The measurement data were analyzed using one-way ANOVA, and LSD test and Mann-Whitney U test were used to compare the mean values between groups. A *P*-value <0.05 was considered statistically significant.

## 3 Result

### 3.1 Preparation and characterization of NPBer nanomedicine

**FIGURE 1:**
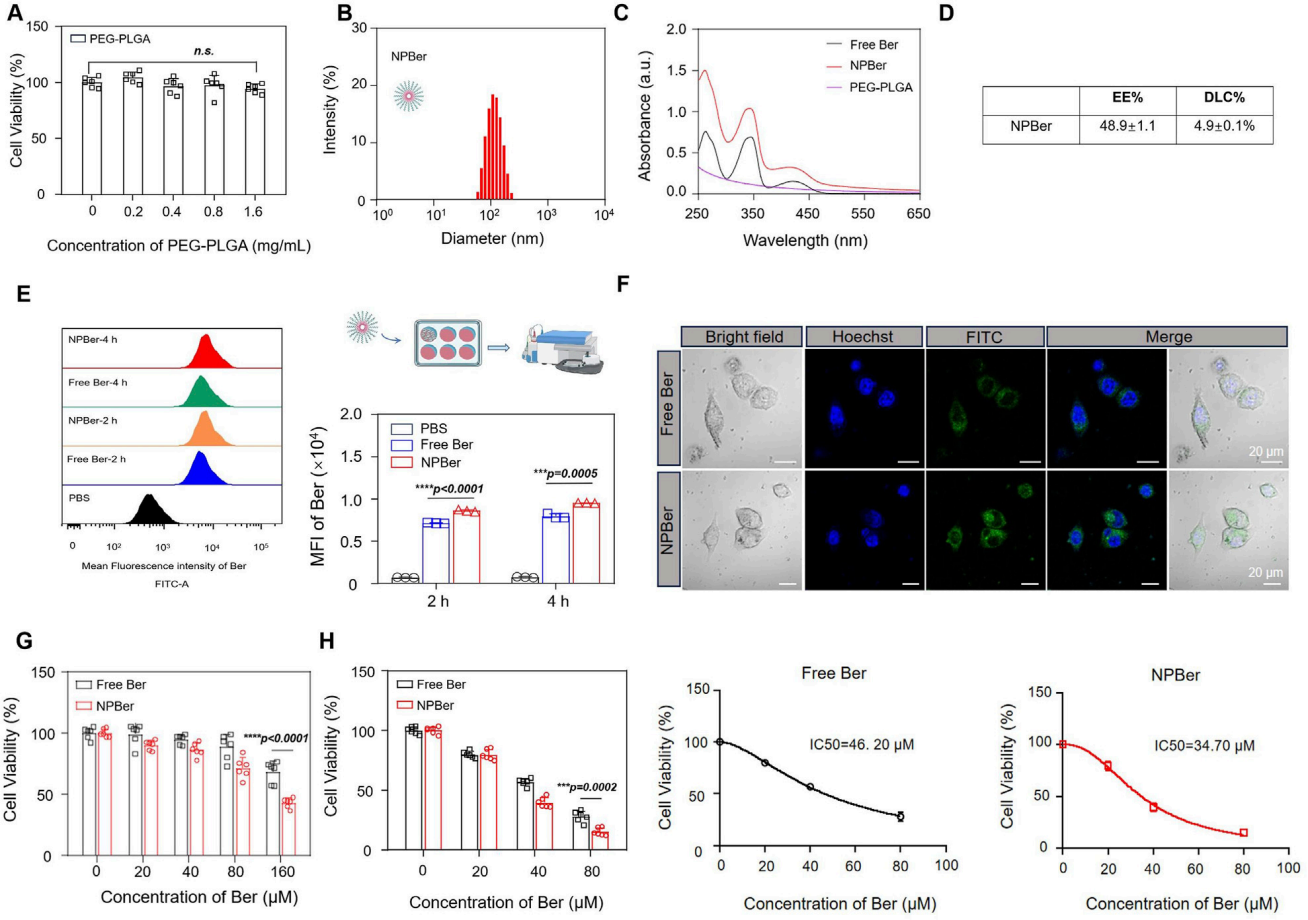
Preparation, Characterization and the intracellular uptake of NPBer, and *In vitro* experiments affect the proliferation of tumor cells. **(A)** Effect of PEG-PLGA on cell activity of HCT116 CRC cells. **(B)** The hydrodynamic size of the NPBer. **(C)** UV−vis spectra of Free Ber, NPBer, and PEG-PLGA. **(D)** The entrapment efficiency (EE) and the drug loading capacity (DLC)of NPBer. **(E)** Flow cytometry analysis of HCT116 CRC cells co-incubated with Free Ber, NPBer, or PBS. **(F)** CLSM images of HCT116 CRC cells internalization of Free Ber or NPBer. Blue: Hoechst 33,342, green: Ber. Scale bar: 20 μm. **(G)** Viability of HCT116 CRC cells were co-cultured with Free Ber and NPBer at different concentrations for 4 h. **(H)**Viability of HCT116 CRC cells were co-cultured with Free Ber and NPBer at different concentrations for 36 h.

First, we assessed the impact of PEG-PLGA nanomaterials on cell viability by testing a concentration range from 0 to 1.6 mg/mL. The results indicated that PEG-PLGA nanomaterials did not affect cell viability (Figure 1A). PEG-PLGA nanoparticles and Ber were synthesized using the nanoprecipitation method. Dynamic light scattering (DLS) analysis revealed that the size of the bare NPBer was approximately 102.3 ± 1.9 nm (Figure 1B). Additionally, UV-Vis spectroscopy demonstrated that NPBer exhibited absorption peaks similar to those of free Berberine (Figure 1C). The entrapment efficiency (EE) of NPBer was found to be 48.9% ± 1.1%, and the drug loading capacity (DLC) of Berberine within NPBer was 4.9% ± 0.1% (Figure 1D).

### 3.2 Cellular uptake and cellur proliferation of NPBer *in vitro*

To investigate the effect of drug-encapsulating nanomaterials on cellular uptake, flow cytometry (FCM) was employed to measure the fluorescence intensity of drug internalization. Compared to Free Ber, NPBer demonstrated significantly higher cellular uptake in HCT116 CRC cells at various time points, indicating that nanomaterial encapsulation enhances Ber’s internalization (Figure 1E). Confocal laser scanning microscopy (CLSM) further confirmed that HCT116 CRC cells incubated with NPBer exhibited superior uptake efficiency compared to cells treated with Free Ber (Figure 1F).

To ascertain whether Ber, when encapsulated with nanomaterials, retains its ability to be swiftly absorbed by cells and sustain its activity, we conducted an experiment. We first incubated various concentrations of Free Ber or NPBer for 4 h. Subsequently, we replaced the medium with fresh complete medium and extended the incubation period for an additional 20 h. Following this, we employed the CCK-8 assay to evaluate the cellular response. The results showed that compared with the Free Ber group, the cell proliferation inhibition ability of the NPBer group was stronger with the increase of drug concentration during early incubation (Figure 1G). These results indicated that NPBer could exhibit stronger antitumor activity, which was consistent with the previous cell uptake experimental results, mainly due to the easier uptake of NPBer by HCT116 CRC cells.

Subsequently, we evaluated the ongoing effect of Free Ber or NPBer on cell proliferation. HCT116 CRC cells were co-cultured with Free Ber or NPBer at different concentrations. The CCK-8 assay results, presented in Figure 1H, revealed that cell viability in the NPBer group was significantly lower than in the Free Ber group. The IC50 values for Free Ber and NPBer were 46.20 μM and 34.70 μM, respectively (Figure 1H).

### 3.3 RNA-seq analysis

**FIGURE 2:**
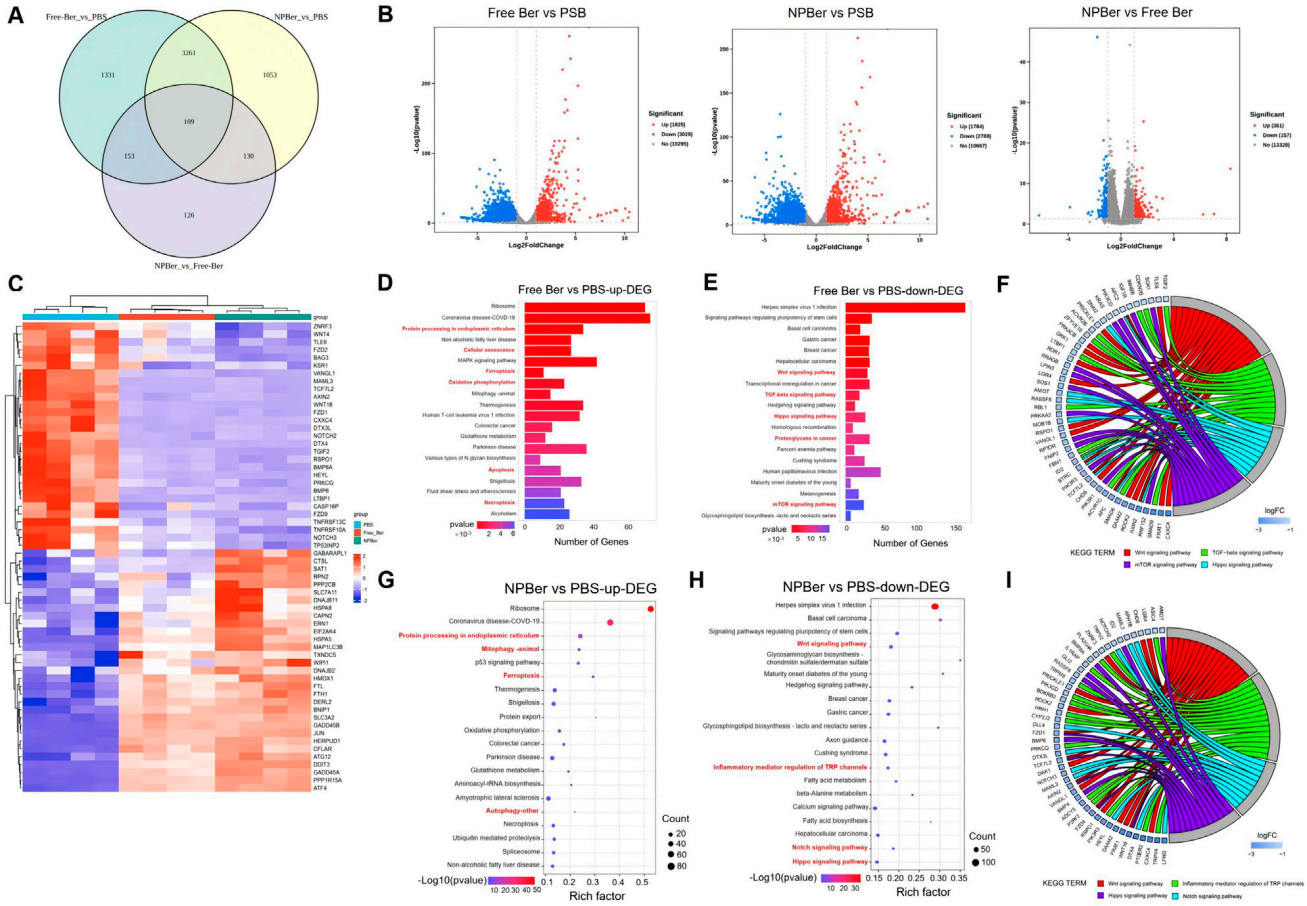
RNA-seq analysis of HCT116 CRC cells treated with PBS, Free Ber and NPBer. **(A)** Venn diagram revealed the number of genes transcribed in each treatment group. **(B)** Volcano plots displayed the differentially expressed genes. **(C)** Heat-map of gene expressions in cells treated with PBS, Free Ber, and NPBer. KEGG analysis of **(D)** upregulated and downregulated **(E)** differentially expressed genes between cells treated with PBS and those treated with Free Ber. **(F)** The chord diagram displays specific molecules/genes enriched in pathways between cells treated with PBS and those treated with Free Ber in KEGG. KEGG analysis of **(G)** upregulated and downregulated **(H)** differentially expressed genes between cells treated with PBS and those treated with NPBer. **(I)** The chord diagram displays specific molecules/genes enriched in pathways between cells treated with PBS and those treated with NPBer in KEGG.

To elucidate the mechanism of NPBer, we performed RNA-Seq analysis on HCT116 CRC cells exposed to PBS, Free Ber, or NPBer. Our initial analysis focused on the transcriptional profiles of 6,163 genes (Figure 2A). As shown in Figure 2B, we identified a distinct set of 1,331 genes that were exclusively transcribed in cells treated with Free Ber compared to the PBS-treated control group. Conversely, 1,053 genes were specifically transcribed in cells treated with NPBer relative to the PBS group. Additionally, 126 genes were uniquely transcribed in NPBer-treated cells compared to those treated with Free Ber.

Figure 2B also illustrates that, compared to the PBS control, Free Ber treatment led to the upregulation of 1,825 genes and downregulation of 3,029 genes. Similarly, NPBer treatment resulted in the upregulation of 1,784 genes and downregulation of 2,769 genes. Notably, NPBer treatment induced differential gene expression compared to Free Ber, with an upregulation of 361 genes and a downregulation of 157 genes.

To visualize these changes, we compared the top 60 differentially expressed genes (DEGs) from each sample and generated a heatmap (Figure 2C). We utilized the Kyoto Encyclopedia of Genes and Genomes (KEGG) database (http://www.genome.jp/) for bio-pathway enrichment analysis to identify significantly enriched pathways among DEGs from the various samples. Figure 2D presents the enrichment analysis results for upregulated genes following Free Ber treatment, highlighting key biological pathways such as protein processing in the endoplasmic reticulum, activation of cellular senescence mechanisms, enhancement of ferroptosis pathways, improved oxidative phosphorylation efficiency, increased apoptosis, and facilitated necroptosis.

Figure 2E shows the enrichment analysis results for downregulated genes after Free Ber treatment, revealing significant downregulation of pathways including Wnt signaling, TGF-beta signaling, Hippo signaling, and mTOR signaling—all of which are closely related to CRC development. Additionally, proteoglycans incancer were found to be downregulated. Figure 2F displays a chord diagram illustrating specific molecules/genes that impact the main enriched pathways in KEGG. These findings collectively reveal the multifaceted effects of Free Ber on the biological processes of CRC cells.

NPBer treatment significantly enhanced the expression levels of specific biological pathways, similar to those observed with Free Ber treatment. This included increased protein processing within the endoplasmic reticulum and augmentation of ferroptosis pathways, along with additional improvements in mitophagy and autophagy (Figure 2G). Conversely, Figure 2H illustrates that NPBer treatment induced downregulation of pathways akin to Free Ber treatment, notably suppressing the Wnt and Hippo signaling pathways while promoting ferroptosis. Additionally, NPBer treatment downregulated the regulatory role of inflammatory mediators on TRP channels and inhibited the Notch signaling pathway. The key molecules and genes involved in these pathways are further detailed in the chord diagram (Figure 2I). These findings highlight the potential of drug encapsulated in nanomaterials to modulate cellular signal transduction and inflammatory responses effectively.

### 3.4 Biodistribution, targeting capacity, antitumor performance and biosafety of NPBer *In Vivo*

**FIGURE 3:**
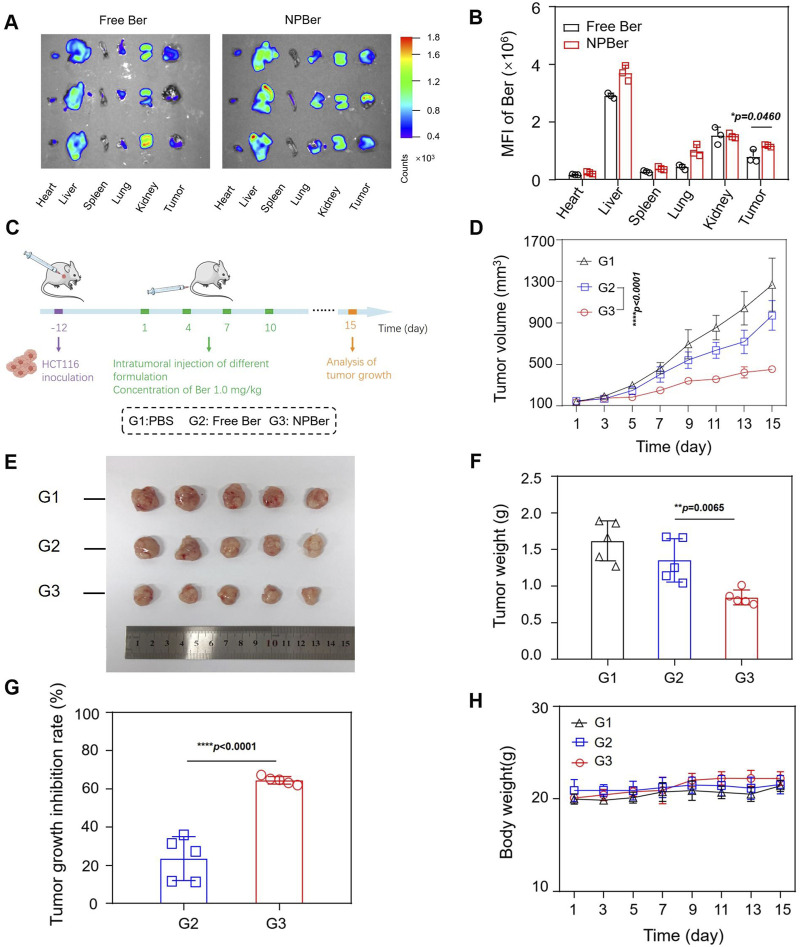
Biodistribution, Targeting Capacity and Antitumor Performance of NPBer *In Vivo*. **(A)** Fluorescence images and **(B)** mean fluorescence intensity of *ex vivo* tumor tissues and major organs. **(C)** Schematic treatment schedule. Arrows show the time points of intravenous injection of different drugs (n = 5). **(D)** HCT116 CRC tumor average growth curve after different treatments. **(E)** Images, weights **(F)** and growth inhibition rate **(G)** of extracted tumor tissues after various treatments. **(H)** The change curve of Mice body weight in various treatment groups.

HCT116 CRC xenograft tumor-bearing mice were established with three mice per group to investigate the *in vivo* biodistribution and targeting capacity of NPBer. Mice received tail vein injections of either Free Ber or NPBer (n = 3 each group), and the fluorescence signals were monitored using an *In Vivo* Imaging System (IVIS). After 24 h, the mice were sacrificed, and ex*vivo* biodistribution of NPBer was analyzed. Figure 3A and B reveal that NPBer treatment resulted in significantly stronger fluorescence at the tumor site compared to Free Ber treatment. In major organs, the fluorescence intensity was most pronounced in the liver, with lower intensity observed in other organs such as the heart, spleen, lungs, and kidneys.

To evaluate the antitumor efficacy of NPBer, HCT116 CRC xenograft tumor-bearing mice were intravenously injected with PBS, Free Ber, or NPBer (at a total dose of 1 mg Ber/kg, n = 5 each group) via the tail vein on days 1, 4, 7, and 10 (Figure 3C). On day 15, blood samples, as well as major tissues and organs, were collected from the treated mice for comprehensive analysis. Figure 3D shows that NPBer treatment led to the most significant suppression of HCT116 tumor growth, compared to Free Ber, which exhibited a moderate inhibitory effect on tumor progression. Assessment of tumor tissue images, post-treatment weight, and tumor growth inhibition rates demonstrated that NPBer was the most effective in terms of anti-tumor efficacy (Figures 3E–G). There were no significant changes in body weight among the mice in any treatment group throughout the study period (Figure 3H).

**FIGURE 4:**
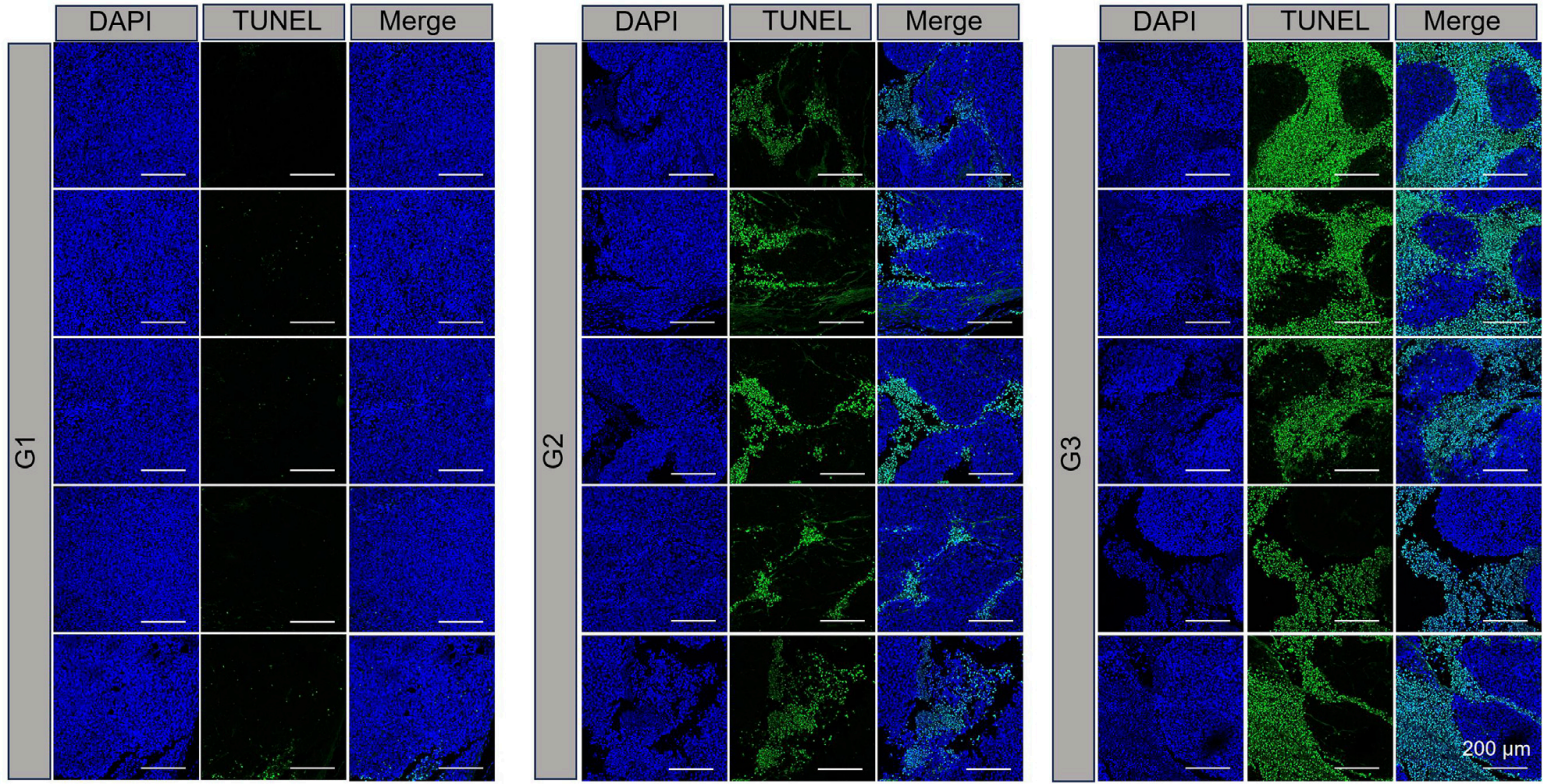
TUNEL staining of tumor tissues. G1: treatment with PBS; G2: treatment with Free Ber; G3: treatment with NPBer.

**FIGURE 5:**
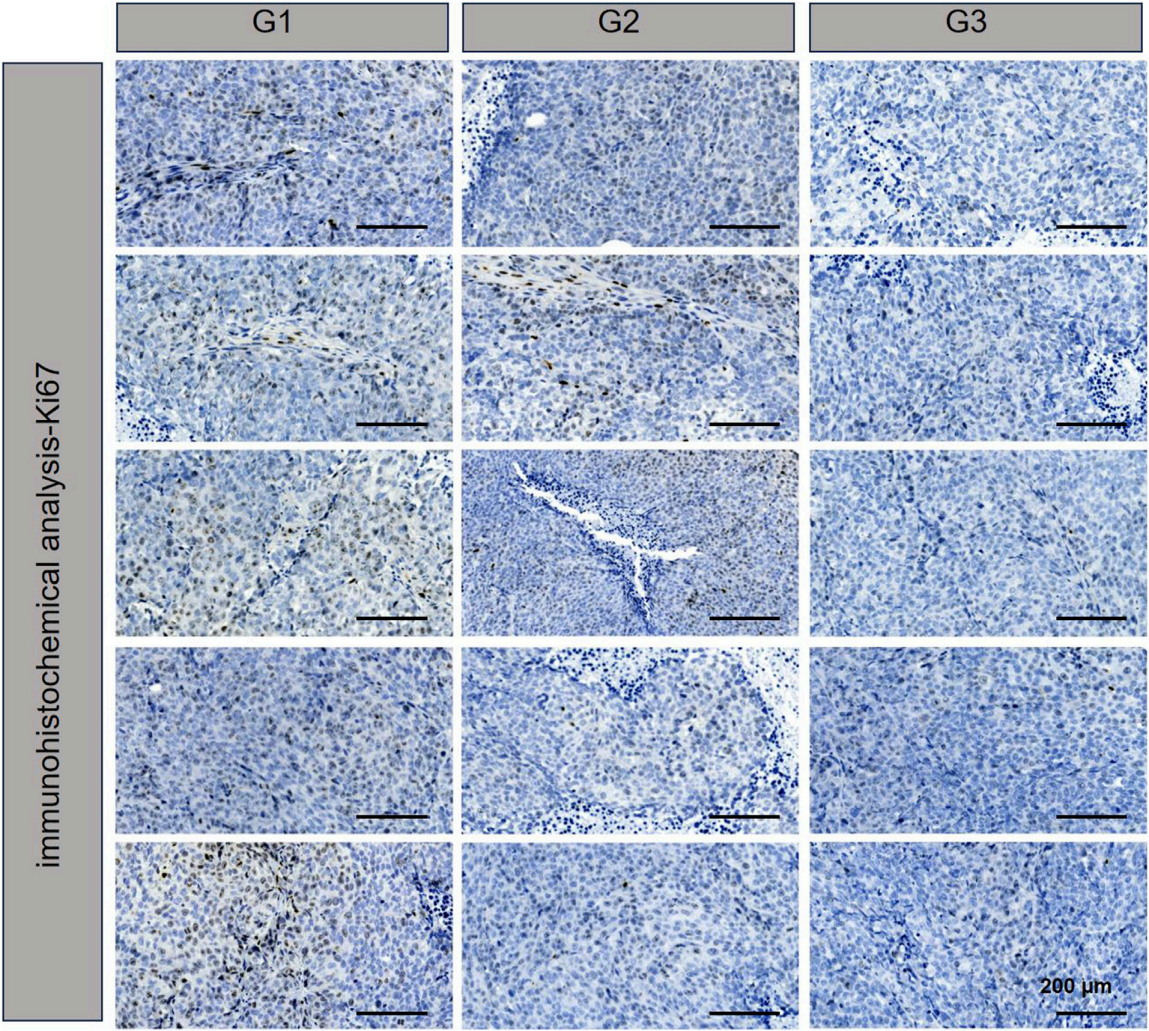
Ki67 of tumor tissues.G1: treatment with PBS; G2: treatment with Free Ber; G3: treatment with NPBer.

TUNEL assay results, which are depicted in Figure 4, revealed DNA double-strand breaks. Nuclei were stained blue with 4′,6-diamidino-2-phenylindole (DAPI), while nuclear damage was indicated by green fluorescence. The proportion of TUNEL-positive cells with green fluorescence was notably higher in the NPBer group, followed by the Free Ber group, compared to the control tissues. Immunohistochemical staining further confirmed that NPBer treatment was the most effective in inhibiting tumor cell proliferation, as evidenced by the significant reduction in brown-stained Ki67-positive cells (Figure 5). These results indicate that NPBer demonstrated superior performance in targeting tumors.

**FIGURE 6:**
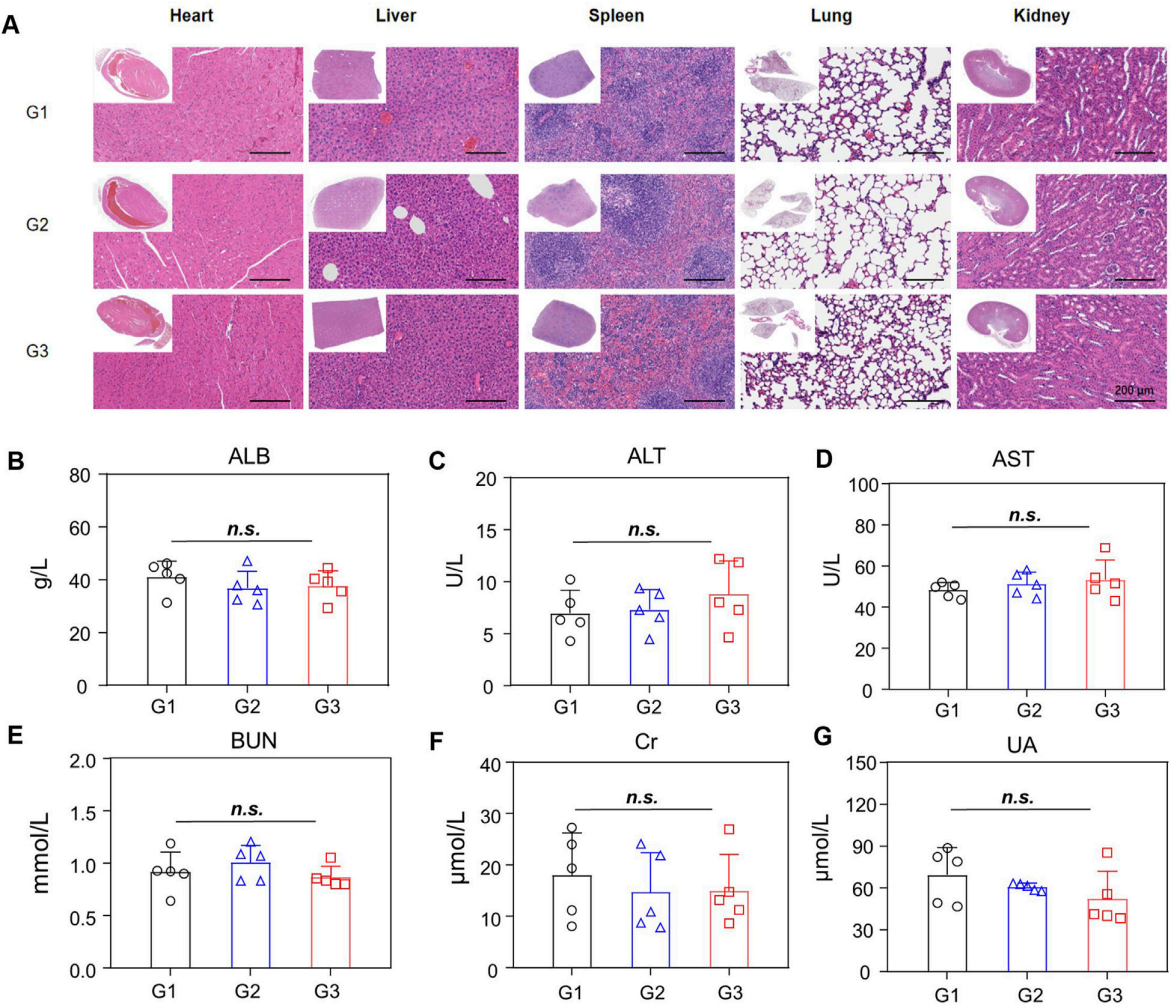
Biosafety of NPBer *In Vivo*. **(A)** The assessment of main organs by H&E staining. **(B–G)** Biochemical analysis of the serum of mice with various treatments (n = 5): **(B)** ALB; **(C)** ALT/GPT; **(D)** AST/GOT; **(E)** BUN; **(F)** Cre; **(G)** UA. G1: treatment with PBS; G2: treatment with Free Ber; G3: treatment with NPBer.

Next, the bio-safety of NPBer was evaluated *in vivo*. Figure 3H illustrated that there were no significant changes in body weight among mice in any treatment group throughout the treatment period. Furthermore, histological examination using hematoxylin and eosin (H&E) staining of major organs revealed no discernible morphological changes in mice treated with Free Ber or NPBer, compared to those treated with PBS (Figure 6A). Additionally, an extensive analysis of blood physiological and biochemical markers showed that levels of albumin (ALB), alanine aminotransferase (ALT), aspartate aminotransferase (AST), blood urea nitrogen (BUN), serum creatinine (Cre), and uric acid (UA) in mice treated with Free Ber or NPBer remained statistically similar to those of the PBS-treated group (Figures 6B–G). Collectively, these findings underscore the minimal toxicity and robust safety profile of NPBer *in vivo*.

## 4 Discussion

In recent years, colorectal cancer (CRC) has emerged as the third most prevalent cause of cancer-related mortality worldwide. This increase is attributed to factors such as an aging population, modern dietary habits, insufficient physical activity, smoking, obesity, and other risk factors. In the United States, CRC is the third leading cause of both new cancer cases and cancer-related deaths among men and women (Siegel et al., 2023). In recent years, significant progress has been made in the third-line treatment options for advanced CRC, with a notable expansion in therapeutic drugs. Notably, the 2020 Chinese Society of Clinical Oncology (CSCO) Guidelines for Diagnosis and Treatment of CRC introduced Trifluridine/Tipiracil (TAS-102), further broadening the therapeutic options. In 2021, the combination therapy of TAS-102 and Bevacizumab gained recognition, offering patients even more diverse treatment choices. However, the challenge remains in achieving specific targeting of antitumor drugs to cancer cells, enhancing the ability of chemotherapy drugs to be taken up by cancer cells, thereby boosting anti-tumor efficacy and mitigating side effects of chemotherapy. In the realm of pharmacotherapy, there is a need to design novel anticancer drugs that can augment therapeutic efficacy, providing CRC patients with both effective treatment modalities and rational therapeutic regimens. Investigating the molecular mechanisms driving CRC initiation and progression, identifying pertinent biomarkers for diagnosis and treatment, and developing innovative therapeutic strategies are essential for improving clinical outcomes, enhancing patients’ quality of life, and extending survival rates. Tumorigenesis and cancer development are typically multifactorial and not attributable to a single gene mutation alone. Consequently, the multi-target properties of active compounds in traditional Chinese medicine (TCM) have increasingly attracted attention as potential anti-tumor agents or adjunctive therapies to conventional chemotherapy (Zhang et al., 2021; Yan et al., 2017).

Berberine (Ber), an isoquinoline alkaloid extracted from Coptis chinensis, has gained considerable attention for its diverse biological activities, including anti-inflammatory, anti-cancer, anti-ulcer, antibacterial, and immune-enhancing effects. These activities are attributed to its pharmacological properties. In the treatment of neurodegenerative diseases, Ber, through its activation of 5′-AMP-activated protein kinase (AMPK), has been shown to counteract common neurodegenerative events (Qin et al., 2020). Recent research, encompassing basic, translational, and clinical studies, has unveiled numerous novel molecular targets of Ber, highlighting its promising potential in treating cardiovascular diseases (Feng et al., 2019). Furthermore, Ber’s anti-tumor properties have recently attracted significant interest. For instance, Xu et al. demonstrated that Ber inhibits gastric cancer development by downregulating IL-6 expression via the JAK2/STAT3 signaling pathway (Xu et al., 2022). Similarly, Gu et al. (2022) proposed that Ber may modulate Protein Arginine Deiminase 4 (PAD4)-associated macrophage functions to prevent lung cancer. Despite these promising findings, the clinical application of Ber is limited by its poor intestinal absorption and rapid metabolism, leading to low oral bio-availability and a short half-life (Wang et al., 2017).

To address these limitations, encapsulating Ber within nanomedicine delivery systems such as PEG-PLGA nanoparticles (NPs) has been explored. Menconi et al. (2021) encapsulated 3PAuCl into biocompatible PLGA-PEG NPs, which preserved the drug’s free characteristics while enhancing apoptosis and mediating autophagy in CRC cells, thereby demonstrating greater cytotoxic effects.Therefore, encapsulating these active substances within the shell of nanomedicine delivery systems such as PEG-PLGA NPs can significantly enhance their performance and effectiveness. These nanoparticles, a recent innovation, exhibit exceptional biocompatibility and lack immunogenicity, thereby boosting the solubility, safety, stability, and targeted delivery of chemotherapeutic agents (Lee et al., 2022; Lu et al., 2024). Research by Paudel et al. found that loading Ber with nanomaterials can enhance its anti-proliferative and anti-metastatic activities against lung cancer cells (Paudel et al., 2022). In these study, due to the enhanced retention and permeation effect, NPs can remain within the tumor tissue-rich blood vessels and the extensive vascular surface. Phytochemicals and their derivatives are swiftly gaining recognition as potential adjunctive treatments for cancer, owing to their ability to modulate signaling cascades that govern cell cycle progression or to directly impact cell cycle regulatory molecules. However, pure phytochemicals have poor bioavailability and short half-lives, making them unsuitable as anticancer drugs. In a related study, Hassani et al. (2022) utilized PLGA-PEG NPs co-loaded with artemisinin and metformin. The results showed that these NPs significantly downregulated hTERT, Bcl-2, cyclin D1, and survivin, while upregulating caspase-3, caspase-7, and Bax in breast cancer cells, thereby enhancing the antitumor therapeutic effect. These findings underscore the potential of combining traditional Chinese medicine (TCM) bioactive components with nanomedicine delivery systems, such as PEG-PLGA NPs, to improve cancer treatment strategies and overcome drug resistance.

Based on the results presented, we hypothesize that encapsulating Ber within nano-drug delivery systems could yield more effective therapeutic outcomes. To investigate this, we first conducted *in vitro* cell viability assays to confirm that pure PEG-PLGA nanomaterials did not affect the viability of CRC cells, thereby demonstrating the biological safety of these materials *in vitro*. Following this, we utilized the PEG-PLGA nano-drug delivery system to encapsulate Ber, resulting in the formation of Berberine nanoparticles (NPBer). Dynamic light scattering (DLS) analysis revealed that NPBer had a size distribution of 102.3 ± 1.9 nm. Ultraviolet-visible (UV-Vis) spectrophotometry was employed to measure the absorption spectra of Free Ber, NPBer, and PEG-PLGA, which indicated that NPBer exhibited the same characteristic UV absorption peak as Free Ber, confirming the successful encapsulation of Ber within the nanoparticles. The encapsulation efficiency of NPBer was determined to be 48.9% ± 1.1%. Additionally, drug loading (4.9% ± 0.1%) was quantified by measuring the absorbance at 340 nm using a multi-function.

To further assess the effect of nano-encapsulated drugs on the uptake capacity of CRC cells, we initially employed flow cytometry to measure fluorescent intensity (MFI). The results indicated that the MFI in the NPBer group was significantly higher compared to the Free Ber group, both at 2 and 4 h post-treatment, with an observable increase in fluorescence over time. Additionally, we performed a confocal laser scanning microscopy (CLSM) experiment. HCT116 CRC cells incubated with NPBer displayed a markedly enhanced intensity of green fluorescence in the cytoplasm compared to the control group. This finding indicates that the nano-encapsulation of Ber notably improves cellular uptake, which is consistent with the flow cytometry results. Further cell proliferation experiments also confirmed that the drug could continue to act on cells after being pretreated for 4 h. Further research on the effect of continuous drug administration (36 h) on cell proliferation showed that compared with the Free Ber group, the cell proliferation in the NPBer group showed a more significant inhibitory effect when the concentration was gradually increased.

Next, we conducted RNA-seq to further explore the mechanisms of how Free Ber and NPBer inhibit CRC tumor progression. After treatment with Free Ber, CRC cells exhibited upregulation of 1,825 genes and downregulation of 3,029 genes. After treatment with NPBer, the expression levels of 1,784 genes were upregulated, while 2,769 genes were downregulated. Gene Ontology analysis of differentially expressed genes revealed that both Free Ber and NPBer significantly impacted the translation process in the cytoplasm, as well as the structural components of cytoplasmic ribosomes and ribosomes. NPBer further enhanced the nuclear transcription mRNA catabolic process and affected intracellular structural composition, particularly the formation and function of ribonucleoprotein complexes, strengthening regulatory capabilities for ubiquitin ligase inhibitor activity and tRNA binding at the molecular function level. Bioinformatic pathway enrichment analysis using the KEGG database showed that Free Ber treatment activated cell senescence mechanisms, enhanced ferroptosis pathways, improved oxidative phosphorylation efficiency, intensified tumor cell apoptosis, and promoted programmed death. Enrichment analysis of downregulated genes after Free Ber treatment identified CRC-related signaling pathways, including Wnt, TGF-beta, Hippo, and mTOR signaling pathways. The Wnt signaling pathway is a complex network of protein interactions, most commonly observed in tissue embryonic development and cancer but also involved in normal adult physiological processes. Multiple reports have elucidated that the classical Wnt signaling pathway is a recognized driver of colon cancer and holds significant therapeutic significance (Wan et al., 2021). Tanton et al. investigated clinical target therapies for Wnt/β-catenin signaling, exploring a novel β-catenin/BCL9 complex inhibitor that can block oncogenic Wnt signaling and disrupt cholesterol homeostasis in colorectal cancer (Tanton et al., 2022). The TGF-beta signaling pathway plays a crucial role in CRC through four primary mechanisms: promoting epithelial-mesenchymal transition (EMT) by activating downstream signaling pathways (such as Smad-dependent and non-Smad-dependent pathways), enhancing angiogenesis by up-regulating angiogenesis-related factors (e.g., VEGF, MMPs, and Ang-2), inhibiting immune cell activation and function to create an immunosuppressive microenvironment, and regulating stem cell properties in CRC by modulating related signaling pathways such as Wnt/β-catenin and Notch (Li et al., 2022). Modifications and disruptions within crucial elements of the Hippo pathway can trigger cancer development, increase malignancy, foster invasion, promote migration, stimulate metastasis, and induce resistance to therapeutic interventions (Mohajan et al., 2021). The mTOR signaling pathway promotes tumor cell survival, proliferation, and cell cycle progression (Silva et al., 2021). KEGG results show that NPBer treatment also enhances the ferroptosis pathway in CRC cells, additionally boosting mitochondrial autophagy and autophagy functions in tumor cells. NPBer treatment also downregulates the regulatory effects of inflammatory mediators on TRP channels and inhibits the Notch signaling pathway. Ion channels hold crucial positions in a variety of biological processes, like cell cycle regulation and the progression of cancer. Specifically, the TRP family of channels has emerged as potential therapeutic targets (Marini et al., 2023). The Notch signaling pathway has been reported to play a key role in the development of CRC, with at least 86% of CRC and 56% of adenoma patients exhibiting gene over-expression in the Notch signaling pathway (Shaik et al., 2020). Our previous research also indicates that mutations in the Notch signaling pathway are related to the enhancement of anti-tumor immunity in CRC (Wang et al., 2020).

To further investigate the therapeutic potential of Ber encapsulated in nanomaterials, we utilized a nude mouse model implanted with CRC cells. Initially, we examined the biodistribution of NPBer *in vivo*. The fluorescence intensity within the tumor region of the NPBer-treated mice was notably higher, confirming that nanoencapsulation improves the drug’s targeting ability. To assess the therapeutic impact, we compared the inhibitory effects of NPBer on CRC tumors with those of Free Ber and PBS. Tumor volumes in both the Free Ber and NPBer groups were significantly reduced compared to the PBS group. Importantly, the NPBer group exhibited significantly smaller tumor volumes compared to the Free Ber group, providing *in vivo* evidence that nano-encapsulation enhances Ber’s efficacy in inhibiting tumor growth. Additionally, TUNEL and Ki67 assays of tumor tissues from each group indicated that NPBer induced maximal tumor cell apoptosis and effectively inhibited cell proliferation. These findings underscore the superior therapeutic potential of Ber when encapsulated in nanomaterials.

To further evaluate the *in vivo* bio-safety of the nano-drug delivery system, we monitored the body weights of nude mice throughout the study and conducted histological and biochemical analyses at the study’s conclusion. The results including Body Weight Analysis: No significant changes in body weight were observed across all treatment groups, indicating no adverse effects on general health. Histological Examination: Hematoxylin and eosin (HE) staining of major organs—including the liver, spleen, heart, lungs, and kidneys—revealed normal cellular morphology. No abnormalities were detected in the nuclei or intracellular structures of these organs. Serum Biochemical Analysis: Serum biochemical parameters did not show any significant deviations among the different groups, suggesting that the nano-drug delivery system did not induce any detectable systemic toxicity. These findings confirm the excellent biosafety profile of the nano-drug delivery materials used in this study.

## 5 Conclusion

This research focused on the synthesis of a novel nanoparticle-encapsulated berberine (NPBer) and evaluated its therapeutic potential. The NPBer nanoparticles demonstrated exceptional stability and durable drug targeting *in vivo*. They were rapidly internalized by tumor cells and effectively inhibited tumor cell proliferation. The activation of Free Ber in CRC cells triggered a cascade of cellular mechanisms, including enhanced ferroptosis, improved oxidative phosphorylation efficiency, and intensified apoptosis, while modulating critical signaling pathways such as Wnt, TGF-beta, Hippo, and mTOR. Furthermore, NPBer facilitated increased mitochondrial and overall autophagy activity, fine-tuned the inflammatory environment by regulating TRP channels, and suppressed the Notch signaling pathway. Collectively, these findings underscore NPBer’s potential as a promising therapeutic approach for CRC and suggest its broader applicability in the treatment of various cancers.

## Data Availability

The datasets presented in this study can be found in online repositories. The names of the repository/repositories and accession number(s) can be found below: https://www.ncbi.nlm.nih.gov, PRJNA1166403.
